# MPBoot: fast phylogenetic maximum parsimony tree inference and bootstrap approximation

**DOI:** 10.1186/s12862-018-1131-3

**Published:** 2018-02-02

**Authors:** Diep Thi Hoang, Le Sy Vinh, Tomáš Flouri, Alexandros Stamatakis, Arndt von Haeseler, Bui Quang Minh

**Affiliations:** 1 University of Engineering and Technology, Vietnam National University, Hanoi, Vietnam; 2 Department of Genetics, Evolution and Environment, University College London, Gower Street, London, WC1E 6BT UK; 3 Heidelberg Institute for Theoretical Studies, Heidelberg, Germany; 4 Karlsruhe Institute of Technology, Institute for Theoretical Informatics, Karlsruhe, Germany; 5 Center for Integrative Bioinformatics Vienna, Max F. Perutz Laboratories, University of Vienna, Medical University Vienna, Campus Vienna Biocenter 5, A-1030 Vienna, Austria; 6 Bioinformatics and Computational Biology, Faculty of Computer Science, University of Vienna, Vienna, Austria

**Keywords:** Phylogenetic inference, Nonparametric bootstrap, Maximum parsimony

## Abstract

**Background:**

The nonparametric bootstrap is widely used to measure the branch support of phylogenetic trees. However, bootstrapping is computationally expensive and remains a bottleneck in phylogenetic analyses. Recently, an ultrafast bootstrap approximation (UFBoot) approach was proposed for maximum likelihood analyses. However, such an approach is still missing for maximum parsimony.

**Results:**

To close this gap we present MPBoot, an adaptation and extension of UFBoot to compute branch supports under the maximum parsimony principle. MPBoot works for both uniform and non-uniform cost matrices. Our analyses on biological DNA and protein showed that under uniform cost matrices, MPBoot runs on average 4.7 (DNA) to 7 times (protein data) (range: 1.2–20.7) faster than the standard parsimony bootstrap implemented in PAUP*; but 1.6 (DNA) to 4.1 times (protein data) slower than the standard bootstrap with a fast search routine in TNT (fast-TNT). However, for non-uniform cost matrices MPBoot is 5 (DNA) to 13 times (protein data) (range:0.3–63.9) faster than fast-TNT. We note that MPBoot achieves better scores more frequently than PAUP* and fast-TNT. However, this effect is less pronounced if an intensive but slower search in TNT is invoked. Moreover, experiments on large-scale simulated data show that while both PAUP* and TNT bootstrap estimates are too conservative, MPBoot bootstrap estimates appear more unbiased.

**Conclusions:**

MPBoot provides an efficient alternative to the standard maximum parsimony bootstrap procedure. It shows favorable performance in terms of run time, the capability of finding a maximum parsimony tree, and high bootstrap accuracy on simulated as well as empirical data sets. MPBoot is easy-to-use, open-source and available at http://www.cibiv.at/software/mpboot.

**Electronic supplementary material:**

The online version of this article (10.1186/s12862-018-1131-3) contains supplementary material, which is available to authorized users.

## Background

Phylogenetic inference on empirical data typically includes bootstrapping. This enables the reconstructed tree to be annotated with support values for each of its branches. The standard non-parametric bootstrap [1, 2] is a popular method in which so-called pseudo-replicates are created by sampling sites from the multiple sequence alignment (MSA), with replacement. For every replicate, a bootstrap tree is reconstructed by conducting an independent search on each bootstrap MSA. The set of bootstrap trees can then be used to build a consensus tree, or map the support values onto the tree inferred from the original MSA [2].

Maximum parsimony (MP) is widely used to infer phylogenies ([3] and references therein). Since calculating the parsimony score is less complex and computationally cheaper than calculating the likelihood of a tree, methods to build MP trees have been applied to large-scale data sets [4, 5]. However, computing the branch support for MP trees is still time consuming especially for large data sets. In addition to run-time limitations, the standard bootstrap is also known to be conservative [6]: the support values estimated by the standard bootstrap often underestimate the probability that a branch is true.

Here, we introduce MPBoot, a novel method for rapidly approximating the MP bootstrap. MPBoot is inspired by the ultrafast bootstrap approximation for maximum likelihood [7]. It employs additional tree search techniques like subtree pruning and regrafting (SPR) and the parsimony ratchet [8]. In the following we present the MPBoot method and a benchmark study that compares MPBoot with the popular TNT [5] and PAUP* [9] programs.

## Methods

### Maximum parsimony principle

Let *A*^*data*^ denote an MSA of *n* sequences and *m* parsimony informative sites. Parsimony informative sites are grouped into site-patterns *D*_1_, *D*_2_, …, *D*_*k*_ with frequencies *d*_1_, *d*_2_, …, *d*_*k*_, respectively. The parsimony score of a tree topology *T* given *A*^*data*^ is calculated as:

1 $$ MP\left(T|{A}^{data}\right)=\sum \limits_{i=1}^k MP\left(T|{D}_i\right)\times {d}_i, $$

where *MP*(*T*| *D*_*i*_) is the parsimony score for tree *T* at site pattern *D*_*i*_.

Given a tree *T*, *MP*(*T*| *D*_*i*_) is computed efficiently using the Fitch algorithm [10] when the costs of change between character states are the same. Although conceptually simple a uniform cost matrix is not biologically meaningful. For example, it is well known that transitions occur more frequently than transversions in DNA sequences; thus it is plausible to assign lower costs to transitions. However, when using a non-uniform cost matrix, one cannot conveniently interpret the parsimony score as the minimum number of substitutions anymore. For a non-uniform cost matrix, the Sankoff algorithm [11] is used to compute *MP*(*T*| *D*_*i*_). A MP tree search aims to find a tree with the minimal parsimony score. Finding *the* best-scoring MP tree is NP-complete [12], thus tree search heuristics are necessary.

### MPBoot

To minimize computing time, the key features of the MPBoot approach are (i) to sample trees from tree space solely based on the original MSA instead of conducting independent tree searches for each bootstrap MSA and (ii) to quickly compute the MP scores of the sampled trees for all bootstrap MSAs. In the following we describe these key components and the overall workflow of MPBoot.

#### Tree sampling on the original MSA

Unlike the standard bootstrap that performs an independent tree search for each bootstrap MSA, MPBoot first generates a set of bootstrap MSAs and then searches through tree space based on the original MSA. Trees encountered during this tree search are considered as potential MP trees for each bootstrap MSA.

The MPBoot tree search on the original MSA works by progressively rectifying a candidate set *C* of distinct, locally optimal trees. To initialize the candidate set, MPBoot constructs 100 locally optimal MP trees by randomized stepwise addition [13] followed by a hill-climbing subtree pruning and regrafting (SPR) search [14]. We sort these trees increasingly by their MP-scores, and then select the first 5 distinct trees to create the initial candidate set. The idea for the candidate set C is inspired by genetic algorithm [15], which maintains a population of trees to preserve the diversity. Throughout tree search, C will be updated continuously with better trees. This completes the *initial step* of MPBoot.

In the subsequent *exploration step*, MPBoot alternates between perturbation of tree topologies and hill-climbing. This is repeated many times in order to move out of local optima in the tree search space.

##### Perturbation

MPBoot first randomly selects a tree *T*_*C*_ from the candidate set *C*. Then *T*_*C*_ is perturbed by either (i) performing a random nearest neighbor interchange (NNI) on 50% of randomly selected inner branches to generate *T*^∗^ or (ii) applying the parsimony ratchet [8]. The parsimony ratchet duplicates 50% of the parsimony informative sites of the original MSA to generate a perturbed MSA. Subsequently, the ratchet performs a hill-climbing SPR search on this perturbed MSA starting from *T*_*C*_ to find a locally optimal tree *T*^∗^. In summary, *T*^∗^ is created either by a tree perturbation (random NNIs) or by an alignment perturbation strategy.

##### Hill-climbing

The tree *T*^∗^ subsequently serves as a starting tree for a hill-climbing SPR search to infer a locally optimal tree *T*^∗∗^for *A*^*data*^. If *MP*(*T*^∗∗^| *A*^*data*^) is smaller than or equal to the largest MP-score on *A*^*data*^ of a tree in the candidate set then *T*^∗∗^ replaces the corresponding tree in the candidate tree set. We call a hill-climbing step successful if *MP*(*T*^∗∗^| *A*^*data*^) is strictly smaller than the smallest MP-score on *A*^*data*^ of a tree in the candidate set. Otherwise, it is unsuccessful, that is, we did not find a better tree.

The exploration step applies the perturbation and successive hill-climbing steps until the last *n*^′^ (rounding up the number of sequences to the nearest hundred) hill-climbing searches were unsuccessful. This allows more thorough search for alignments with many sequences. Hence, MPBoot stops because it is unlikely to find a better tree. The exploration step is completed.

#### Resampling parsimony score (REPS)

For a tree, *T,* encountered during the hill-climbing SPR search, MPBoot computes its score for each bootstrap MSA, *A*^*bootstrap*^, and then updates the bootstrap tree for *A*^*bootstrap*^ if *T* shows a better MP score. Since this is time consuming, we need to efficiently calculate the MP scores of *T* for *A*^*bootstrap*^. To this end, we adapted the resampling estimated log-likelihoods method introduced by Kishino et al. [16] to calculate the *resampling parsimony score* (REPS) for each bootstrap MSA. However, while resampling estimated log-likelihoods only yields *approximate* log-likelihoods, REPS always returns the *exact* parsimony score. For a tree *T* and the site-pattern scores *MP*(*T*| *D*_*i*_) computed from *A*^*data*^, the parsimony score for *A*^*bootstrap*^ is quickly calculated as the weighted sum of site-pattern parsimony scores:

2 $$ MP\left(T|{A}^{bootstrap}\right)=\sum \limits_{i=1}^k MP\left(T|{D}_i\right)\times {d}_i^{bootstrap}, $$

where the ${d}_i^{bootstrap}$ are the resampling frequencies of the patterns *D*_*i*_ in *A*^*bootstrap*^. Thus, it is not necessary to recompute the parsimony score for each site pattern, bootstrap replicate and tree.

#### Speeding up REPS computation

To further accelerate MP-score computations, MPBoot utilizes two additional algorithmic optimizations.

First, we introduce a threshold *MP*_*max*_, such that the parsimony scores for the bootstrap MSAs are evaluated using Eq. (2) only for those trees *T,* found in the hill-climbing step, for which *MP*(*T*| *A*^*data*^) < *MP*_*max*_ holds. Initially *MP*_*max*_ = ∞. After the first hill-climbing step, we set *MP*_*max*_ to the lower 10%-quantile of the MP-score distribution on the original MSA for all trees found in the hill-climbing step. In the subsequent hill-climbing steps, we only consider trees that have an MP-score for the *A*^*data*^ smaller than *MP*_*max*_. The MP-scores of these trees form the distribution, which is then used to update *MP*_*max*_ after every hill-climbing step as above.

Second, we abort REPS computations for an *A*^*bootstrap*^ if we cannot expect that the *MP*(*T*| *A*^*bootstrap*^) will be smaller than the most parsimonious score *MP*_*best*_(*A*^*bootstrap*^) found so far. To this end, we sort the site patterns *D*_*i*_ in descending order of their MP scores based on the first tree constructed in the initial step. The theoretically smallest MP score, *MP*_*min*_(*D*_*i*_),is equal to the number of distinct character states in *D*_*i*_ minus 1 for uniform cost matrices. For non-uniform cost matrices, *MP*_*min*_(*D*_*i*_) is equal to the length of the minimum spanning tree on the cost graph, where nodes correspond to character states and edge weights to the substitution cost between states. We stop the REPS computation if

3 $$ \sum \limits_{i=1}^j MP\left(T|{D}_i\right)\times {d}_i^{bootstrap}+\sum \limits_{i=j+1}^k{MP}_{min}\left({D}_i\right)\times {d}_i^{bootstrap}>{MP}_{best}\left({A}^{bootstrap}\right) $$

for some index *j* (1 ≤ *j* ≤ *k*), because *T* cannot be an MP tree for *A*^*bootstrap*^. The first partial sum on the left hand side in (3) is the MP score computed for the first *j* site-patterns of *A*^*bootstrap*^, whereas the second partial sum is the lower bound of the MP score for the remaining *k* − *j* site-patterns. If inequality (3) holds, we know that *T* is worse than the currently best bootstrap tree for *A*^*bootstrap*^, without having to compute the MP score of the remaining *k* − *j* site-patterns.

#### MPBoot workflow

We can now summarize the MPBoot workflow:

0) Input: an MSA *A*^*data*^ with *n* sequences (taxa).
1) Initial step: Generate *B* bootstrap MSAs, *A*_1_, *A*_2_, …, *A*_*B*_. For each *A*_*b*_ initialize the bootstrap tree *T*_*b*_ ≔ *null* and *MP*(*T*_*b*_| *A*_*b*_) ≔  + ∞. Initialize a set of trees *S* ≔ {} and the threshold *MP*_*max*_ ≔  + ∞. Initialize the candidate set for *A*^*data*^ as explained in 2.2.1.
2) Exploration step: Perform the perturbation and hill-climbing steps on a randomly selected tree from the candidate set for *A*^*data*^, as explained in 2.2.1. Every time a new tree, *T*, with *MP*(*T*| *A*^*data*^) < *MP*_*max*_ is encountered, add T to S and compute *MP*(*T*| *A*_*b*_), for *b* = 1, …, *B* based on Eqs. (2) and (3). If *MP*(*T*| *A*_*b*_) < *MP*(*T*_*b*_| *A*_*b*_), update *T*_*b*_ ≔ *T*. When the hill-climbing step is completed, update *MP*_*max*_ as the lower 10%-quantile of the MP-scores for trees in *S*.
3) Stopping rule: If the last *n*^′^ hill-climbing steps were unsuccessful, go to 4. Otherwise, go back to 2.
4) Refinement step: For each MP-tree *T*_*b*_ (*b* = 1, …, *B*) and the corresponding MSA *A*_*b*_ do a hill-climbing SPR search and replace *T*_*b*_ by the new MP tree, if a better parsimony score is found.
5) Summary step: Construct a consensus tree from the bootstrap trees {*T*_1_, *T*_2_, …, *T*_*B*_} , or map the support values onto the best MP tree that was found for *A*^*data*^.

#### Implementation details

MPBoot uses the phylogenetic likelihood library (PLL) [17] for efficient parsimony computations. To reduce the computational cost of SPR searches, the PLL employs a radius that restricts the maximum number of nodes between the subtree pruning and regrafting branches. Thus, we tested two versions denoted by MPBoot SPR3 and MPBoot SPR6, which have an SPR radius of 3 and 6, respectively. Finally, all the core parsimony and REPS calculations are vectorized with Streaming SIMD Extensions (SSE) and Advanced Vector Extensions (AVX): vector intrinsics and bit-wise population count intrinsics that can use the respective hardware features on modern × 86 architectures.

### Data and performance analysis

We compared the performance of MPBoot SPR3 and SPR6 (compiled with SSE4) with the standard bootstrap (1000 replicates), implemented in TNT version 1.1 (October 2014) and PAUP* version 4.0a152 (January 2017). All methods keep a single best tree per bootstrap replicate. We compared DNA and protein alignments using uniform and non-uniform cost matrices. For DNA data the assumed non-uniform cost matrix has a transition cost of 1 and a transversion cost of 2. For protein data the cost of change between two amino acids is defined as the minimum number of nucleotide changes necessary to turn one amino acid into the other amino acid. The resulting cost matrix is modified so as not to violate the triangle inequality [18].

We employed two tree search routines in TNT, namely the *fast* (P. Goloboff, personal communication) and *intensive* search. The fast-TNT uses the command “*mult = rep 1 hold 1” (*i.e.*,* TNT performs a randomized stepwise addition followed by a full tree bisection and reconnection (TBR)) for tree searches on the original and bootstrap MSAs. The intensive-TNT applies the command “*xmult = notarget hits 3 level 0 chklevel +1 1”* for the original MSA and “*mult = rep 1 hold 1”* for the bootstrap MSAs. The *xmult* command combines different search strategies such as the ratchet, sectorial searches, tree fusing, and tree drifting [5]. Thus, intensive-TNT searches the tree space more thoroughly than fast-TNT for the original MSA, but uses the same search strategy as fast-TNT for the bootstrap MSAs.

We also examined the standard bootstrap implemented in PAUP* by applying a randomized stepwise addition followed by full TBR searches independently on the original as well as bootstrap MSAs. Due to excessive execution times we could only run PAUP* for the uniform cost matrix. The exact TNT and PAUP* commands are included in the Additional file 1.

#### Simulated data

To assess computing time, capability of finding an MP-tree and the accuracy of the bootstrap estimates, we repeated the simulations for DNA and protein alignments described in [7]. In brief, we downloaded MSAs from the PANDIT database [19], selected the best-fit ML models and inferred the ML tree for each MSA. These inferred trees were then treated as true trees to simulate MSAs under the best-fit model parameters, with the same length and gap patterns as the original PANDIT MSAs. It should be noted that a parsimony analysis violates the assumptions of the selected best-fit models. We excluded 15 DNA and 17 protein MSAs where TNT or PAUP* runs did not finish. Hence, the simulated data comprised 6207 DNA MSAs with 4 to 403 (median: 10) sequences (DNA-PANDIT) and 6165 protein MSAs with 4 to 374 (median: 10) sequences (AA-PANDIT).

#### Real data

To benchmark MPBoot we reanalyzed the 115 TreeBASE MSAs analyzed by Nguyen et al. [20], which comprised 70 DNA MSAs with 201 to 767 (median: 233) sequences and 45 protein MSAs with 50 to 194 (median: 78) sequences. However, we had to exclude M9915 because intensive-TNT did not converge. All summary statistics are thus based on the remaining 114 MSAs.

## Results

### Computing times

To obtain an overall runtime ranking among the examined bootstrap methods, we compare their accumulated running times for the 114 TreeBASE MSAs (Table 1). Under the uniform cost matrix, fast-TNT needed 14.9 h and is the fastest method, followed by MPBoot SPR3 (36.2 h) and SPR6 (70.1 h). PAUP* is the slowest method (206.1 h; about 14 times slower than fast-TNT). However, under non-uniform costs MPBoot SPR3 is the fastest program (202 h), with MPBoot SPR6 being the runner-up (491 h).

**Table 1:** Cumulative runtimes (hours) for the five tested methods for 114 TreeBASE MSAs

	Uniform cost	Non-uniform cost
fast-TNT	**14.9**	1784
MPBoot SPR3	36.2	**202**
MPBoot SPR6	70.1	491
intensive-TNT	72.5	2470
PAUP*	206.1	NA

Run times in bold-face highlight the respective fastest method under the given cost models

**Fig. 1:**
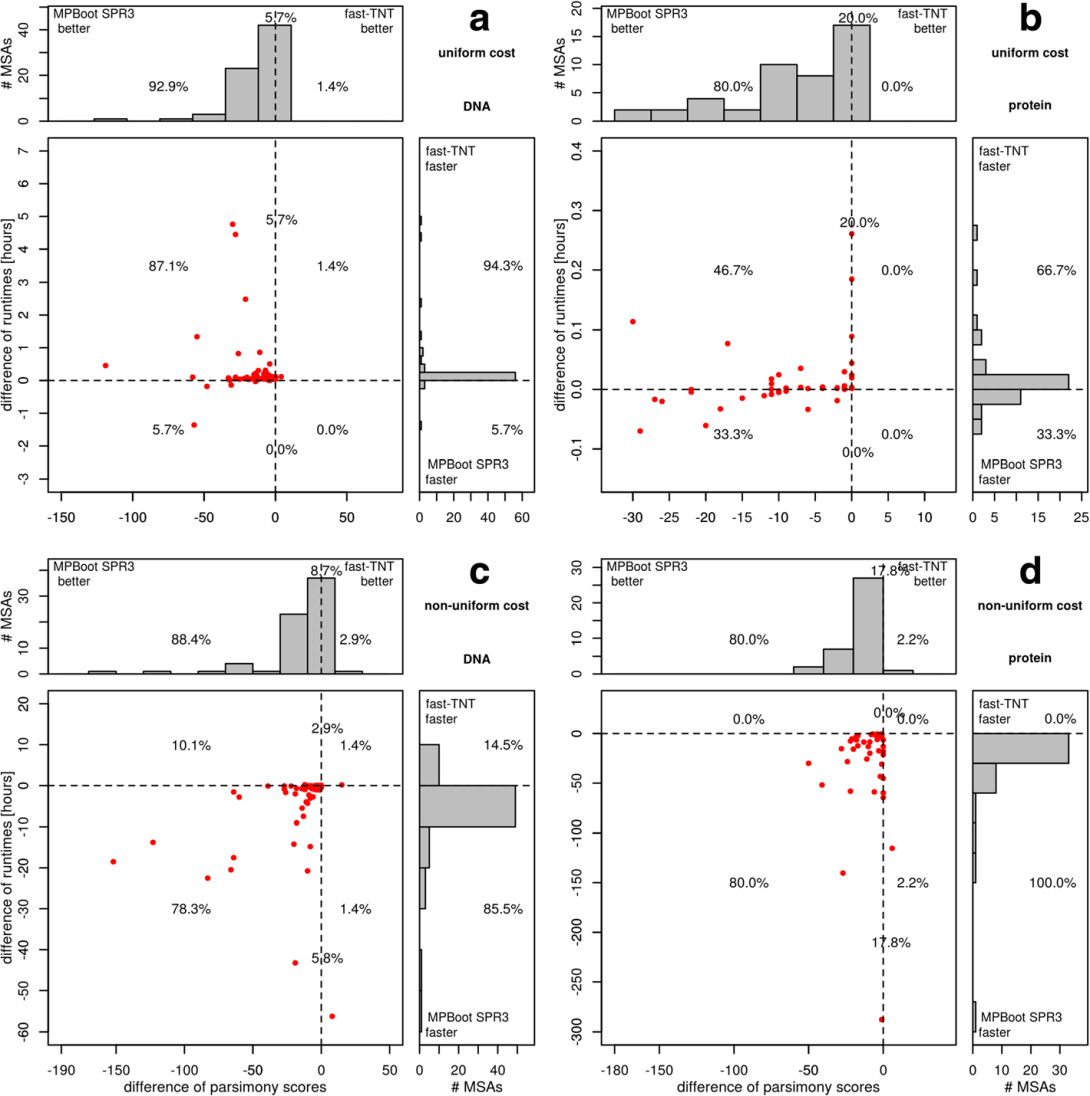
Performance comparison in terms of runtimes and MP scores between MPBoot SPR3 and fast-TNT under uniform (**a**, **b**) and non-uniform cost matrices (**c**, **d**), for real DNA and amino-acid MSAs. Each dot in the main diagrams represents a single MSA. The y-axis displays the difference between the CPU times of the two programs. The x-axis displays the difference between parsimony scores of the MP trees on the original MSA inferred by the two programs. The histograms at the top and the side present the marginal frequencies. Dots to the left of the vertical dashed line represent alignments where MPBoot found a better parsimony score. If a dot is below the horizontal dashed line, the bootstrap analysis by MPBoot was faster. Percentages in the quadrants of histograms denote the fraction of alignments in that region. Percentages on the dashed line reflect the number of alignments where two programs obtain equal MP scores

**Fig. 2:**
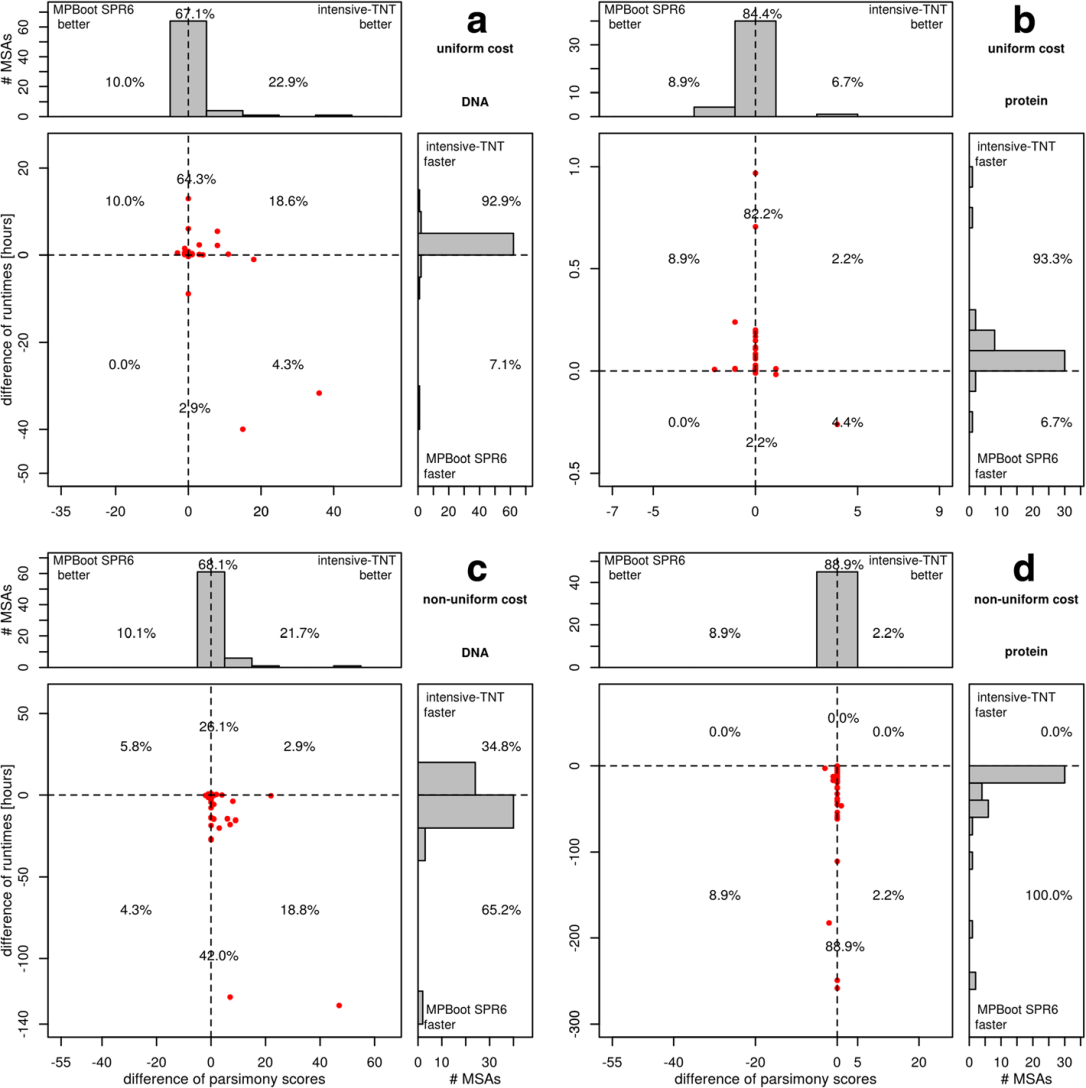
Performance comparison in terms of runtimes and MP scores between MPBoot SPR6 and intensive-TNT under uniform (**a**, **b**) and non-uniform cost matrices (**c**, **d**), for real DNA and amino-acid MSAs. Each dot in the main diagrams represents a single MSA. The y-axis displays the difference between the CPU times of the two programs. The x-axis displays the difference between parsimony scores of the MP trees on the original MSA inferred by the two programs. The histograms at the top and the side present the marginal frequencies. Dots to the left of the vertical dashed line represent alignments where MPBoot found a better parsimony score. If a dot is below the horizontal dashed line, the bootstrap analysis by MPBoot was faster. Percentages in the quadrants of histograms denote the fraction of alignments in that region. Percentages on the dashed line reflect the number of alignments where two programs obtain equal MP scores

Instead of focusing on the accumulated run-times it is instructive to study the distribution of runtime differences across all MSAs. Figure 1 shows the comparison between fast-TNT and MPBoot SPR3. Here, fast-TNT is faster than MPBoot SPR3 for uniform cost matrix (94.3% DNA and 66.7% protein MSAs) but took substantially much more time than MPBoot SPR3 under non-uniform cost matrix (up to 55 and 280 h for DNA and protein, respectively). Similar performance advantages are observed for the comparison between intensive-TNT and MPBoot SPR6 methods (Fig. 2).

### Capability of finding the best-known MP scores

**Fig. 3:**
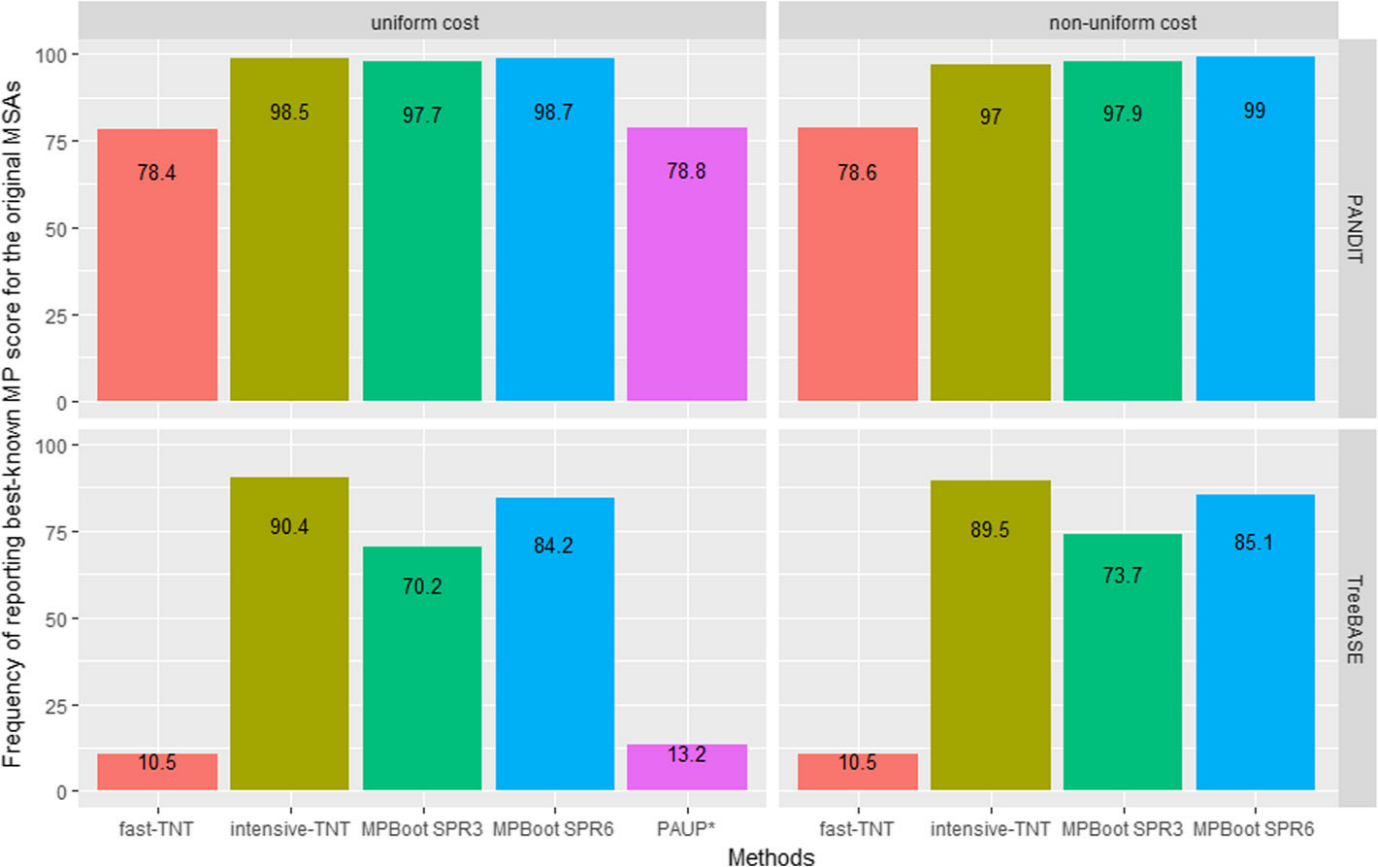
Performance of tested methods in the inference of MP trees for the original MSAs. The bar-plots show the frequencies with which each of the five tested methods produced the best MP score for original MSAs in the (**a**) simulated PANDIT and (**b**) TreeBASE data sets. Note that the best MP score for a given MSA can be found by more than one methods; therefore the sum of frequencies for a data set may be greater than one. Data for PAUP* under the non-uniform cost matrix is not available due to excessive execution times

Because the speedups of MPBoot may impede our ability to find the best MP scores on the original MSAs, we compared the best MP scores obtained by different methods for each original MSA (both simulated and real data). To this end, we calculated the frequency with which each method obtained the smallest score among the five tested methods over all MSAs (Fig. 3). Please note that for the analysis MP scores of trees output by MPBoot and TNT were recomputed by PAUP*.

For simulated data (Fig. 3; upper plots), fast-TNT and PAUP* show similar frequencies of finding the best-known scores (75% to 82%). This is not surprising because they implemented similar search strategies. MPBoot SPR3, MPBoot SPR6 and intensive-TNT achieve higher frequencies of finding the best-known scores (95% to 99.5%).

Further analysis of 114 TreeBASE MSAs shows reduced frequencies for all methods (Fig. 3; lower plots; Additional file 2). Notably, the frequencies of obtaining the best-known scores for fast-TNT and PAUP* drop to 10% - 13%, whereas MPBoot and intensive-TNT obtain moderate (70% - 85%) and high (90%) frequency, respectively. We also observed that the results do not differ between uniform and non-uniform cost analyses.

While Fig. 3 only shows the frequencies of obtaining the best scores, it is more informative to assess for each method how much the inferred scores deviate from the best scores. To do so, we compared MPBoot SPR3 and fast-TNT in terms of the differences in MP scores for each TreeBASE MSA (Fig. 1; each dot corresponds to one MSA). For uniform cost matrix MPBoot SPR3 found lower scores than fast-TNT for 92.9% DNA (Fig. 1a) and 80% protein MSAs (Fig. 1b). Similar results are observed for non-uniform cost matrix (Fig. 1c and d). However, intensive-TNT showed better scores than MPBoot SPR6 for DNA (Fig. 2a and c) and similar performance for protein MSAs (Fig. 2b and d).

### Accuracy of bootstrap estimates

We compared the *accuracy* of estimated bootstrap support values for MPBoot with the standard bootstrap implemented in fast-TNT, intensive-TNT, and PAUP*. Here, the accuracy of a method, M, is defined by *f*_M_(*x*), the proportion of all branches with support value x% (across all reconstructed trees) that occur in the true tree [6]. *f*_M_(*x*) reflects the probability that a branch with support *x*% is a true branch. Method M is called unbiased if *f*_M_(*x*) = *x*% for all values of *x*. If the curve of *f*_M_(*x*) is above the diagonal line then the bootstrap method underestimates the probability of a branch being true (i.e., the method is conservative). Otherwise (the curve for *f*_M_(*x*) is below the diagonal), the method overestimates the probability that a branch is true.

**Fig. 4:**
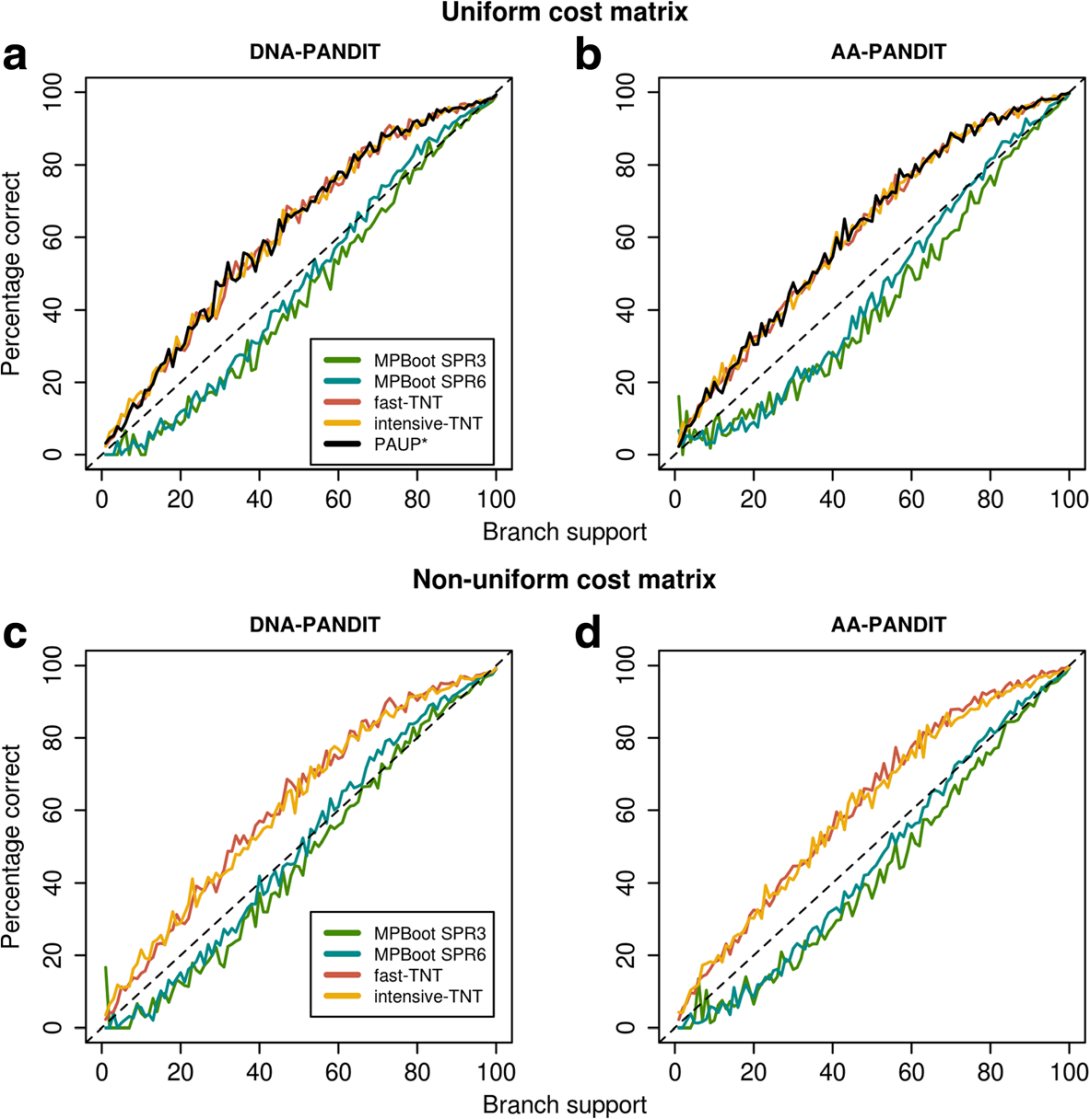
Accuracy of bootstrap supports on simulated PANDIT DNA and protein MSAs for MPBoot SPR3 (green curves), MPBoot SPR6 (blue curves), fast-TNT (red curves), intensive-TNT (yellow curves), and PAUP* (black curves) under uniform cost matrices (**a**, **b**) and non-uniform cost matrices (**c**, **d**). The bin size on x-axis is 1%

Figure 4a and b show the accuracy functions for the five methods under uniform cost matrices on the simulated data. It shows that the data type (nucleotides or amino acids) of alignments does not influence the accuracy of the bootstrap estimates. TNT methods and PAUP* underestimate the probability of branches being true (Fig. 4a and b; curves above the diagonal). For example, a branch with a PAUP* support value of at least 80% has more than a 95% probability of being true. This corroborates previous studies (e.g., [6]) that the standard bootstrap is conservative. MPBoot SPR6 obtained almost unbiased branch supports, at least for branch supports> 70% (Fig. 4a and b; curves close to the diagonal). This allows for a more intuitive interpretation of bootstrap support values. That is, to achieve a true probability of 95%, MPBoot SPR6 support values need to be 95%.

Similarly, we observe that assuming non-uniform cost matrices, MPBoot SPR6 is less conservative than fast-TNT for branch supports> 70% (Fig. 4c and d; PAUP* was not run due to excessive computing times).

To further investigate the discrepancy between the bootstrap support estimates of MPBoot and standard bootstrap, we compared the MP scores of bootstrap trees obtained by MPBoot and TNT, assuming the uniform cost matrix. The bootstrap MP scores by MPBoot SPR3 were only 2.7 substitutions (median; score difference range: − 60.8 to 100.5) higher than those by fast-TNT. Whereas MPBoot SPR6 achieved bootstrap MP scores of 1 substitution (median; score difference range: − 63.4 to 28.9) lower than fast-TNT (Additional file 3; Additional file 4: Figure S1). Overall, we did not observe any substantial difference between scores of bootstrap trees obtained by MPBoot and TNT.

## Discussion

We present a novel tree search algorithm for maximum parsimony tree inference and a fast approximation for the parsimony bootstrap (MPBoot), which is inspired by the UFBoot approach [7] for maximum likelihood tree inference. MPBoot differs from UFBoot in four aspects: (i) it computes an exact (instead of an approximate) computation of bootstrap MP scores, (ii) it uses SPR moves instead of NNI moves in the hill-climbing search, (iii) it includes the application of the parsimony ratchet, (iv) it applies a refinement step to improve the scores of the most parsimonious bootstrap trees obtained in the exploration phase using the bootstrap MSAs. We show that this combination leads to an efficient exploration of parsimony tree space. Compared with the standard bootstrap implemented in PAUP* and TNT, MPBoot shows good performance both in terms of bootstrap accuracy and run time on simulated as well as empirical data sets. Compared with TNT for tree searches on the original MSAs, MPBoot obtains MP scores that are better than those of fast-TNT and comparable to intensive-TNT. For the bootstrap analysis MPBoot yielded bootstrap MP scores comparable to those of TNT.

It was shown that the conservative behavior of the standard bootstrap is attributed with the so-called *rogue taxa* [21–23], whose positions may vary in the tree without changing the MP scores (i.e., low phylogenetic signals). We conjecture that the less conservative behavior of MPBoot (Fig. 4) likely resulted from reduced effect of rogue taxa as follows. On average, one third of original MSA sites are not sampled in bootstrap MSAs, leading to the more pronounced effect of rogue taxa when tree search is performed independently for each bootstrap MSA as in standard bootstrap. In contrast, MPBoot performs tree search on the original MSA (with a small refinement step at the end), reducing the effect of rogue taxa because MP scores are computed on all original MSA sites. At the same time MPBoot still achieved bootstrap trees with comparable bootstrap MP scores as TNT. It will be interesting, but beyond the scope of this study, to compare the bootstrap tree topologies obtained by MPBoot and TNT to potentially identify rogue taxa and thus to understand the more unbiased behavior of MPBoot.

The trade-off between MP scores and run times is noticeable when comparing between fast-TNT and intensive-TNT or between MPBoot SPR3 and MPBoot SPR6. Note that, MPBoot SPR6 sometimes does not find better trees than MPBoot SPR3 although one would expect this due to the more thorough search, however the stochastic nature of tree search cannot guarantee a consistently better performance. Although fast-TNT and PAUP* use the same tree search strategy, fast-TNT is substantially faster than PAUP* because of the highly optimized code in TNT. MPBoot could potentially be further accelerated by additional code optimization and implementation of additional efficient computational operations [4]. MPBoot is slower than TNT under a uniform cost matrix but faster under a non-uniform cost matrix. This advantage of MPBoot over TNT is due to the fact that efficient computational operations in TNT rely on bit-wise operations. However, the majority of these operations cannot be applied to non-uniform cost matrices. Besides, assuming a non-uniform cost matrix, computing the parsimony score for a tree on a bootstrap MSA while finding the corresponding MP bootstrap tree (as in any standard bootstrap implementation) is considerably more expensive than applying Eq. 2 (as in MPBoot implementation).

We also compared the computing time between MPBoot and the ultrafast bootstrap for maximum likelihood (UFBoot). MPBoot is about 1.2 and 8.6 time faster than UFBoot2 [24] on DNA and protein MSAs, respectively (Additional file 4: Figure S2).

For support values > 70%, MPBoot SPR3 slightly overestimates the probability of a branch being true for protein MSAs but is sufficiently accurate for DNA MSAs (Fig. 4; green curves). MPBoot SPR6 provides almost unbiased bootstrap estimates for protein MSAs but slightly underestimates the probability of a branch being true for DNA MSAs for support values > 70% (Fig. 4; blue curves). Thus, to obtain a direct interpretation of bootstrap support values, users are advised to apply MPBoot SPR6. We note that, the support values obtained by MPBoot tend to be higher than those inferred by PAUP*, fast-TNT, and intensive-TNT; they are thus not directly comparable. For example, a branch with a fast-TNT support of 70–80% may show an MPBoot support of 95%, which roughly corresponds to 95% chance of being correct.

MPBoot approximates bootstrap trees in conjunction with sampling trees for the original MSA. Each tree encountered during the tree search on the original MSA will be immediately evaluated for all bootstrap MSAs. The stopping rule is to determine a reasonable number of hill-climbing steps for finding the best tree on the original MSA. Hence, even if the search stops prematurely, all bootstrap MSAs are always examined.

We provide an option to save equally optimal trees per bootstrap replicate in MPBoot. Using this option we observed that, for each TreeBASE MSA, 85% of the bootstrap replicates that have equally optimal trees before the refinement step, get refined into the same bootstrap tree after the refinement. Since we only collect trees as bootstrap trees after the refinement step, this option does not change the accuracy of MPBoot but only induces a higher computational cost. Therefore, by default MPBoot only keeps one best bootstrap tree per bootstrap MSA. Moreover, the refinement step is essential. Otherwise, MPBoot tends to provide over-confident support values.

In this study, all examined bootstrap methods keep a single tree per bootstrap MSA and use the same method (split frequencies) to summarize bootstrap trees. However, we should note that, MPBoot/PAUP* and TNT use different approaches to summarize bootstrap trees if multiple trees are kept per bootstrap MSA. MPBoot and PAUP* first assigns a weight that is equal to the reciprocal of the number of equally parsimonious trees found in the bootstrap replicate to each bootstrap tree. Then, they compute bootstrap support values. TNT first computes a strict consensus for the equally parsimonious trees found for each bootstrap replicate. Thereafter, it calculates bootstrap support values from these strict consensus trees [25]. Other methods are also available for summarizing bootstrap trees such as the GC method [25].

The running time of all bootstrap methods is mainly determined by the number of pseudoreplicates. Intensive-TNT becomes faster than MPBoot when a lower number of pseudoreplicates is used. For example, intensive-TNT and MPBoot become faster by factors of 7.3 and 1.8 respectively, when executed with 100, instead of 1000 pseudoreplicates on real datasets under uniform cost matrices. Although biologists might only execute 100 pseudoreplicates to reduce the time to completion of their analyses, theoretical studies recommend using several thousands of pseudoreplicates to obtain highly reliable results [26]. Pattengale et al. [27] asserted that the required number of bootstrap replicates is highly data-dependent. Based on real data analyses they conclude that Hedges formula [26] provides a reasonable upper bound for the number of required replicates.

We also examined the performance of MPBoot with SPR radii larger than 6 and found that increasing the radius produced slightly shorter trees but incurred higher computational cost. Furthermore, MPBoot SPR6 obtained bootstrap trees with MP scores comparable to or sometimes even better than TNT, which implements a TBR search. This suggests that MPBoot SPR6 performs well for the search on the original MSA as well as on the bootstrap MSAs. Nixon [8] found that the ratchet percentages between 5% and 25% worked well in his analysis of parsimony scores on the original MSA. However, our analysis on real data showed that a ratchet percentage of 50% gave best parsimony scores. Thus, we set the SPR radius to 6 and the ratchet percentage to 50% as default in MPBoot. Nevertheless, users have the possibility to change these parameters, if necessary. We used the 10th percentile in determining *MP*_*max*_ after having examined the accuracy and runtime of lower and higher percentile values. On the simulated data, 10th percentile shows best balance for both.

In the future, we plan to increase the flexibility of the MPBoot search by implementing TBR tree rearrangements and the parsimony jackknife [28]. Another aspect of future work is to parallelize MPBoot. Here, the REPS computation can be done for each bootstrap MSA independently, and hence concurrently. For tree searches on the original MSA, one can either parallelize the parsimony score computation over the MSA sites using a shared-memory scheme or distribute distinct independent search iterations to different CPUs [29].

## Conclusions

This paper presents MPBoot, a method for efficient MP tree search and efficiently approximating the standard MP bootstrap. We compared MPBoot with the implementation of the standard MP bootstrap in TNT and PAUP* assuming different cost matrices. MPBoot found MP scores better than fast-TNT and PAUP* and comparable to intensive-TNT. MPBoot SPR6 yields almost unbiased support values regardless of the nature of data and the specific cost matrix. MPBoot also requires substantially shorter run times than PAUP*. An efficient and easy-to-use implementation of MPBoot is freely (open-source under GNU General Public License) available at http://www.cibiv.at/software/mpboot with precompiled binaries for Mac OSX and Linux.

## Additional files

Additional file 1 Commands used in this study for performing parsimony analyses by TNT and PAUP*. (DOCX 14 kb)

Additional file 2 Description of data: MP-score and runtimes (in seconds) of examined methods on 114 out of 115 TreeBASE MSAs. The file contains two sheets: one for results of analyses under uniform cost matrices and the other for those under non-uniform cost matrices. (XLSX 29 kb)

Additional file 3 Spreadsheet of summary statistics for comparing MPBoot and TNT bootstrap tree MP-scores by replicate counts and by MP-score difference for 114 TreeBASE MSAs. (XLS 51 kb)

Additional file 4 Figures for i) distribution of MP-score difference between TNT and MPBoot on bootstrap MSAs for 114 TreeBASE MSAs, ii) distribution of runtime ratio between MPBoot and UFBoot2 for 114 TreeBASE MSAs. (DOCX 169 kb)

## Abbreviations

MP: Maximum Parsimony
MSA: Multiple sequence alignment
NNI: Nearest neighbor interchange
REPS: Resampling parsimony score
SPR: Subtree-pruning and regrafting
SSE: Streaming SIMD Extensions
TBR: Tree bisection and reconnection

## References

[1] Efron B (1979). Bootstrap Methods: another look at the jackknife. Ann. Stat. Institute of Mathematical Statistics.

[2] Felsenstein J (1985). Confidence limits on phylogenies : an approach using the bootstrap. Evolution.

[3] Felsenstein J (2004). Inferring phylogenies.

[4] Goloboff PA (1996). Methods for faster parsimony analysis. Cladistics..

[5] Goloboff PA, Farris JS, Nixon KC (2008). TNT, a free program for phylogenetic analysis. Cladistics..

[6] Hillis DM, Bull JJ (1993). An empirical test of bootstrapping as a method for assessing confidence in phylogenetic analysis. Syst Biol.

[7] Minh BQ, Nguyen MAT, von Haeseler A (2013). Ultrafast approximation for phylogenetic bootstrap. Mol. Biol. Evol..

[8] Nixon KC (1999). The parsimony ratchet, a new method for rapid parsimony analysis. Cladistics..

[9] Swofford DL (2002). PAUP*. Phylogenetic analysis using parsimony (*and other methods). Version 4.

[10] Fitch WM (1971). Toward defining the course of evolution: minimum change for a specific tree topology. Syst Zool.

[11] Sankoff D (1975). Minimal mutation trees of sequences. SIAM J Appl Math.

[12] Graham RL, Foulds LR (1982). Unlikelihood that minimal phylogenies for a realistic biological study can be constructed in reasonable computational time. Math Biosci.

[13] Wagner WH (1961). Problems in the classification of ferns. Recent Adv. Bot. Univ. of Toronto press Toronto, Can UnderwritCanada.

[14] Stamatakis A, Hoover P, Rougemont J, Renner S (2008). A rapid bootstrap algorithm for the RAxML web servers. Syst Biol.

[15] Holland JH (1975). Adaptation in natural and artificial systems.

[16] Kishino H, Miyata T, Hasegawa M (1990). Maximum likelihood inference of protein phylogeny and the origin of chloroplasts. J. Mol. Evol..

[17] Flouri T, Izquierdo-Carrasco F, Darriba D, Aberer AJ, Nguyen L-T, Minh BQ (2015). The phylogenetic likelihood library. Syst Biol.

[18] Letter WWC (1993). To the editor: the triangle inequality and character analysis. Mol Biol Evol.

[19] Whelan S, de Bakker PIW, Quevillon E, Rodriguez N, Goldman N (2006). PANDIT: an evolution-centric database of protein and associated nucleotide domains with inferred trees. Nucleic Acids Res.

[20] Nguyen L-T, Schmidt HA, von Haeseler A, Minh BQ (2015). IQ-TREE: a fast and effective stochastic algorithm for estimating maximum-likelihood phylogenies. Mol. Biol. Evol..

[21] Wilkinson M (1996). Majority-rule reduced consensus trees and their use in bootstrapping. Mol. Biol. Evol..

[22] Aberer AJ, Krompass D, Stamatakis A (2013). Pruning rogue taxa improves phylogenetic accuracy: an efficient algorithm and webservice. Syst Biol.

[23] Lemoine F, Domelevo Entfellner J-B, Wilkinson E, de Oliveira T, Gascuel O (2017). Boosting Felsenstein phylogenetic bootstrap. bioRxiv.

[24] Hoang DT, Chernomor O, von Haeseler A, Minh BQ, Le SV (2017). UFBoot2: improving the ultrafast bootstrap approximation. Mol Biol Evol.

[25] Goloboff PA, Farris JS, Källersjö M, Oxelman B, Ramírez MJ, Szumik CA (2003). Improvements to resampling measures of group support. Cladistics.

[26] Hedges SB (1992). The number of replications needed for accurate estimation of the bootstrap P value in phylogenetic studies. Mol Biol Evol United States.

[27] Pattengale ND, Alipour M, Bininda-Emonds ORP, Moret BME, Stamatakis A (2010). How many bootstrap replicates are necessary?. J Comput Biol United States.

[28] Farris JS, Albert VA, Källersjö M, Lipscomb D, Kluge AG (1996). Parsimony jackknifing outperforms neighbor-joining. Cladistics. Blackwell Publishing Ltd.

[29] Minh BQ, Vinh LS, von Haeseler A, Schmidt HA (2005). pIQPNNI: parallel reconstruction of large maximum likelihood phylogenies. Bioinformatics.

