# Supplementary material for: MPBoot: fast phylogenetic maximum parsimony tree inference and bootstrap approximation
Source: BMC Evol Biol. 2018 Feb 2;18:11. doi: 10.1186/s12862-018-1131-3 (PMC5796505; doi:10.1186/s12862-018-1131-3)
Supplement: Supplementary file 1 — Commands used in this study for performing parsimony analyses by TNT and PAUP*. (DOCX 14 kb) [file 12862_2018_1131_MOESM1_ESM.docx]

## TNT – script for the fast-TNT bootstrap under uniform cost matrix

This bootstrap analysis generates 1000 bootstrap replicates and uses the fast search strategy on both the original MSA and the bootstrap MSAs.

In a Linux machine, to perform the analysis on a DNA alignment example.fa of fasta format, save the following script as fastboot.run, put it in the same directory as the tnt executable, then run the command line:

**./tnt fastboot example.fa dna,**

For protein data, replace “dna” in the last argument of the command line by “prot”.

When the program finishes, you will see three output files.

example.fa.fast.log contains the program messages during the run.

example.fa.fast.best containing the best tree inferred on the original MSA.

example.fa.fast.boottrees containing two bootstrap concensus trees and 1000 bootstrap trees.

| macro=;  mxram 1000;  taxname +100;  taxname=;  log %1.fast.log;  collapse 0;  report =;  watch =;  nstates %2;  nstates nogaps;  p &%1;  hold 2000;  rseed 0;  mult= rep 1 hold 1;  export - %1.fast.best;  resample boot rep 1000 freq savetrees [mult = rep 1 hold 1;];  export - %1.fast.boottrees;  log/;  proc/;  z; |
| --- |

## TNT – script for the intensive-TNT bootstrap under uniform cost matrix

This bootstrap analysis generates 1000 bootstrap replicates and uses the intensive search strategy on the original MSA and the fast search strategy on bootstrap MSAs.

Similar to the instructions for fast-TNT, save the following script as intensiveboot.run, then run the command-line:

**./tnt intensiveboot example.fa dna,**

| macro=;  log %1.intensive.log;  report =;  watch =;  nstates %2;  nstates nogaps;  mxram 1000;  taxname +100;  taxname=;  proc &%1; sect: slack 100;  hold 10000;  rseed 0;  collapse 0;  xmult = notarget hits 3 level 0 chklevel +1 1;  export - %1.intensive.best;  resample boot rep 1000 freq savetrees [mult=rep 1 hold 1];  export - %1.intensive.boottrees;  log/;  proc/;  z; |
| --- |

## TNT – the commands for non-uniform cost matrix

To perform bootstrap analyses for DNA under non-uniform cost matrix with fast-TNT or intensive-TNT, insert to the first blank line in the corresponding script for uniform cost matrix provided above with the following commands for defining cost matrix:

| smatrix =1 (g1ts1tv2) a/c 2 a/g 1 a/t 2 c/g 2 c/t 1 g/t 2;  smatrix +1 .;  ccode -( .; |
| --- |

For protein data, we use the following matrix:

| smatrix =1 (aa_nt_changes) A/R 2 A/N 2 A/D 1 A/C 2 A/Q 2 A/E 1 A/G 1 A/H 2 A/I 2 A/L 2 A/K 2 A/M 2 A/F 2 A/P 1 A/S 1 A/T 1 A/W 2 A/Y 2 A/V 1 R/N 2 R/D 2 R/C 1 R/Q 1 R/E 2 R/G 1 R/H 1 R/I 1 R/L 1 R/K 1 R/M 1 R/F 2 R/P 1 R/S 1 R/T 1 R/W 1 R/Y 2 R/V 2 N/D 1 N/C 2 N/Q 2 N/E 2 N/G 2 N/H 1 N/I 1 N/L 2 N/K 1 N/M 2 N/F 2 N/P 2 N/S 1 N/T 1 N/W 2 N/Y 1 N/V 2 D/C 2 D/Q 2 D/E 1 D/G 1 D/H 1 D/I 2 D/L 2 D/K 2 D/M 2 D/F 2 D/P 2 D/S 2 D/T 2 D/W 2 D/Y 1 D/V 1 C/Q 2 C/E 2 C/G 1 C/H 2 C/I 2 C/L 2 C/K 2 C/M 2 C/F 1 C/P 2 C/S 1 C/T 2 C/W 1 C/Y 1 C/V 2 Q/E 1 Q/G 2 Q/H 1 Q/I 2 Q/L 1 Q/K 1 Q/M 2 Q/F 2 Q/P 1 Q/S 2 Q/T 2 Q/W 2 Q/Y 2 Q/V 2 E/G 1 E/H 2 E/I 2 E/L 2 E/K 1 E/M 2 E/F 2 E/P 2 E/S 2 E/T 2 E/W 2 E/Y 2 E/V 1 G/H 2 G/I 2 G/L 2 G/K 2 G/M 2 G/F 2 G/P 2 G/S 1 G/T 2 G/W 1 G/Y 2 G/V 1 H/I 2 H/L 1 H/K 2 H/M 2 H/F 2 H/P 1 H/S 2 H/T 2 H/W 2 H/Y 1 H/V 2 I/L 1 I/K 1 I/M 1 I/F 1 I/P 2 I/S 1 I/T 1 I/W 2 I/Y 2 I/V 1 L/K 2 L/M 1 L/F 1 L/P 1 L/S 1 L/T 2 L/W 1 L/Y 2 L/V 1 K/M 1 K/F 2 K/P 2 K/S 2 K/T 1 K/W 2 K/Y 2 K/V 2 M/F 2 M/P 2 M/S 2 M/T 1 M/W 2 M/Y 3 M/V 1 F/P 2 F/S 1 F/T 2 F/W 2 F/Y 1 F/V 1 P/S 1 P/T 1 P/W 2 P/Y 2 P/V 2 S/T 1 S/W 1 S/Y 1 S/V 2 T/W 2 T/Y 2 T/V 2 W/Y 2 W/V 2 Y/V 2 ;  smatrix +1 .;  ccode -( .; |
| --- |

## PAUP* – the bootstrap command that uses same strategy as fast-TNT for uniform cost matrix

| bootstrap nreps=1000 search=heuristic format=Phylip replace=yes/ addseq=random nreps=1 swap=tbr multrees=no hold=1 reconlimit=Infinity |
| --- |
