# Supplementary material for: MPBoot: fast phylogenetic maximum parsimony tree inference and bootstrap approximation
Source: BMC Evol Biol. 2018 Feb 2;18:11. doi: 10.1186/s12862-018-1131-3 (PMC5796505; doi:10.1186/s12862-018-1131-3)
Supplement: Supplementary file 4 — Figures for i) distribution of MP-score difference between TNT and MPBoot on bootstrap MSAs for 114 TreeBASE MSAs, ii) distribution of runtime ratio between MPBoot and UFBoot2 for 114 TreeBASE MSAs. (DOCX 169 kb) [file 12862_2018_1131_MOESM4_ESM.docx]

**Supplementary figures**


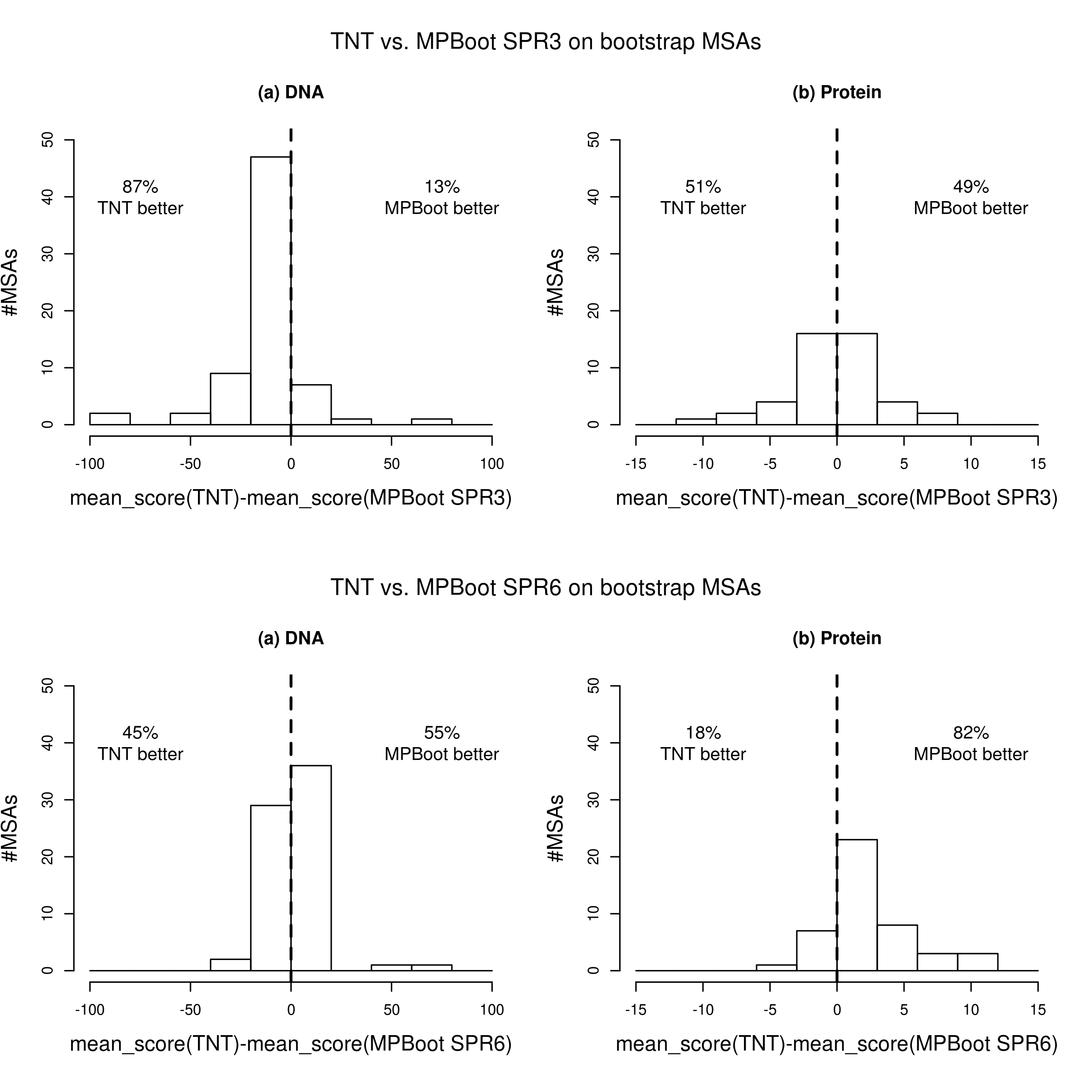


**Figure S1.** Distributions of mean score difference between bootstrap trees by TNT and MPBoot SPR3 (upper) and between TNT and MPBoot SPR6 (lower) for TreeBASE DNA (a) and protein (b) MSAs.


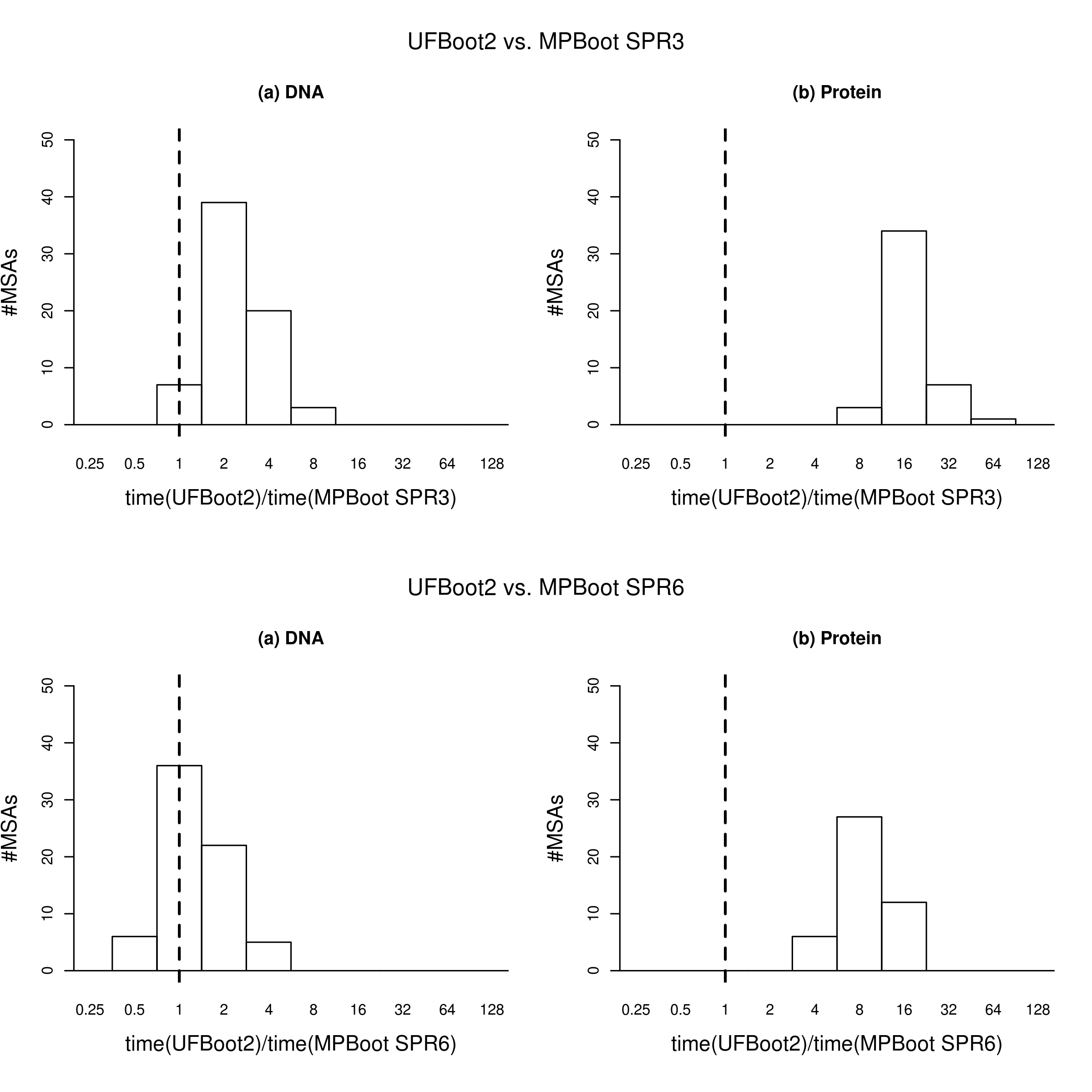


**Figure S2.** Distributions of runtime ratios between UFBoot2 and MPBoot SPR3 (upper) and between UFBoot2 and MPBoot SPR6 (lower) for TreeBASE DNA (a) and protein (b) MSAs.
